# Prevalence of Acute Coronary Syndrome in Patients Suspected for Pulmonary Embolism or Acute Aortic Syndrome: Rationale for the Triple Rule-Out Concept

**DOI:** 10.14740/jocmr2197w

**Published:** 2015-06-09

**Authors:** Saad Al Qahtani, Ahmed Y. Kandeel, Stephane Breault, Anne-Marie Jouannic, Salah D. Qanadli

**Affiliations:** a Department of Radiology & Medical Imaging, Armed Forces Hospital, Southern Region, Saudi Arabia; b Cardio-Thoracic and Vascular Unit, Department of Radiology, University Hospital of Lausanne, Switzerland; c Quantitative Medical Imaging Laboratory, Department of Radiology, University Hospital of Lausanne, Switzerland

**Keywords:** Acute coronary syndrome, Acute aortic syndrome, Pulmonary embolism, Triple rule-out

## Abstract

**Background:**

The aims of the study were to evaluate the prevalence of acute coronary syndrome (ACS) among patients presenting with atypical chest pain who are evaluated for acute aortic syndrome (AAS) or pulmonary embolism (PE) with computed tomoangiography (CTA) and discuss the rationale for the use of triple rule-out (TRO) protocol for triaging these patients.

**Methods:**

This study is a retrospective analysis of patients presenting with atypical chest pain and evaluated with thoracic (CTA), for suspicion of AAS/PE. Two physicians reviewed patient files for demographic characteristics, initial CT and final clinical diagnosis. Patients were classified according to CTA finding into AAS, PE and other diagnoses and according to final clinical diagnosis into AAS, PE, ACS and other diagnoses.

**Results:**

Four hundred and sixty-seven patients were evaluated: 396 (84.8%) patients for clinical suspicion of PE and 71 (15.2%) patients for suspicion of AAS. The prevalence of ACS and AAS was low among the PE patients: 5.5% and 0.5% respectively (P = 0.0001), while the prevalence of ACS and PE was 18.3% and 5.6% among AAS patients (P = 0.14 and P = 0.34 respectively).

**Conclusion:**

The prevalence of ACS and AAS among patients suspected clinically of having PE is limited while the prevalence of ACS and PE among patients suspected clinically of having AAS is significant. Accordingly patients suspected for PE could be evaluated with dedicated PE CTA while those suspected for AAS should still be triaged using TRO protocol.

## Introduction

Patients who present to the emergency department (ED) with chest pain constitute a common and important diagnostic challenge. In the Centers for Disease Control and Prevention survey, evaluation of acute chest pain and related symptoms was the second most common reason for a visit to the ED by a female adult and the most common reason by a male adult in the United States [1-3]. Chest pain accounted for more than 8 million of the ED visits [2, 3] and for about 2 million hospital admissions per year [4].

Among the most clinically relevant conditions causing chest pain that have to be differentiated in the ED are pulmonary embolism (PE), acute aortic syndromes (AAS), and acute coronary syndrome (ACS). The last condition is identified in approximately 15-25% of patients with acute chest pain who are evaluated in EDs [5]. Unfortunately, the number of patients with manifestations of acute myocardial infarction who are inappropriately discharged from the ED is not negligible, about 2-5% of patients [6, 7].

Patients in whom the diagnosis of ACS is missed tend to be younger and to have an atypical presentation and a non-diagnostic electrocardiogram (ECG) [8]. The missed diagnosis of ACS is a common reason for litigation against ED physicians and accounts for up to 25% of the total malpractice liability of ED physicians [9]. On the other hand, uncertainty in the diagnosis of ACS results in the practice of defensive medicine and begets an increased number of diagnostic tests and hospital admissions [10]. The cost of negative inpatient cardiac evaluations is estimated at $6 billion in the United States each year [11].

The one-step CT examination for chest pain, the so-called “triple rule-out (TRO) protocol” used to diagnose ACS, PE, and AAS is increasingly being performed in many institutions equipped with 64-slice or higher multi-detector CT scanners. Although there have been many articles dealing with TRO, these studies did not provide detailed data of delayed diagnosis in assessing patients with atypical chest pain who do not undergo immediate cardiac computed tomoangiography (CTA) [4, 11-19]. In addition, there is limited information regarding overall concepts, such as how to choose the optimal examination (i.e., dedicated coronary CTA versus TRO versus dedicated PE or aortic CTA) based on the specific clinical presentation to the ED [4]. Moreover, the actual incidence of ACS, PE and AAS and the overlap between the three entities in patients presenting to ED with atypical chest pain remains unclear. There are no enough studies describing the overlap between the ACS from a side and the PE/AAS from another side.

The aims of this study were to evaluate the prevalence of ACS among patients with atypical chest pain who are evaluated for AAS or PE with CTA and accordingly discuss the rationale for the use of TRO protocol for triaging these patients.

## Materials and Methods

The study consisted of retrospective review of all consecutive outpatients who presented with atypical chest pain and underwent thoracic CTA, for clinical suspicion of PE and/or AAS, during a consecutive 12 months period at our University Hospital. The hospital’s institutional review board and ethical committee approved the study.

### Eligibility criteria

All patients 18 years or older who presented with a clinical history of non-traumatic atypical or non-angina acute chest pain, suggestive of AAS or PE and who underwent thoracic CTA were included. Definition of atypical or non-angina chest pain was based on the guidelines recently reported by the National Institute of Clinical Excellence (NICE) [20]. Typical angina combines the three following criteria: 1) constricting discomfort in the front of the chest or the neck, shoulders, jaw or arms, 2) precipitated by physical exercise and 3) relieved by rest or glyceryl trinitrate (GTN) within about 5 min. Atypical chest pain is defined as pain with two of the three criteria and non-angina pain with one or none of the three criteria.

Clinical features suggestive of AAS included chest pain, nausea, and diaphoresis, extreme apprehension with a sense of impending doom, associated focal neurologic symptoms or peripheral arterial ischemia. Clinical features suggestive of PE included chest pain, associated shortness of breath, and dizziness. We used the pre-test score to stratify patients on low, intermediate and high probability of PE. Patients with low/intermediate probability and positive D-dimer test as well as patients at high probability were indication for thoracic CTA [21].

### Exclusion criteria

By design, patients without chest pain and those with clinical ECG (i.e. ST elevation or depression of more than 1 mm or T-wave inversion in more than two anatomically continuous leads) or laboratory evidence (elevation of cardiac biomarkers) suggestive of ACS and patients with history of ACS were also excluded from the study.

### Data analysis

Two physicians reviewed in consensus the patients’ files for demographic characteristics, initial or suspected clinical diagnosis (pre-test diagnosis), CT diagnosis and the final diagnosis (post-test diagnosis). The final diagnosis was established using the discharge diagnosis and/or 30 days patient outcome.

Patients were then classified according to their CT finding into AAS, PE or other diagnoses and according to their final diagnosis as having AAS, PE, ACS or other diagnoses.

Quantitative variables were expressed as means ± standard deviations and categorical variables percentages. We calculated the percentage of patients who had a final diagnosis of either PE or AAS (post-test diagnosis), among the patients with an initial suspicion of PE or AAS (pre-test diagnosis). The percentage of patients with a final diagnosis of ACS in each of the previous two groups, PE or AAS, was also calculated as well as among the whole population with atypical chest pain.

The Pearson’s Chi-square (X^2^) test was used to evaluate whether the results were statistically significant or not. A P value < 0.05 was considered significant.

## Results

Four hundred and sixty-seven (467) patients (249 male and 218 female) presenting with atypical chest pain met the eligibility criteria for inclusion in the study. They included 396 (84.8%) patients (mean age 62.3 years) with initial clinical suspicion of having PE (pre-test PE group) and 71 (15.2%) patients (mean age 58.1 years) initially suspected of having AAS (pre-test AAS group).

The final diagnosis (post-test) of the 467 patients in the study included 95 patients with PE, nine patients with AAS, 35 patients with ACS and 328 patients with other diagnosis. The initial clinical suspicion of the 95 patients with final diagnosis of PE included 91 patients (96%) with PE (pre-test PE) and four patients (4%) with AAS (pre-test AAS) while the initial clinical suspicion of the nine patients with AAS included seven patients (77%) with AAS (pre-test AAS) and two patients (22%) with PE (pre-test PE) (Table 1).

**Table 1:** Incidence of PE, AAS and ACS Among Patients With Initial Clinical Suspicion of PE or AAS

Pre-test diagnosis	Post-test diagnosis			
	PE	AAS	ACS	Others
PE (n = 396)	91 (23%)	2 (0.5%)	22 (5.5%)	281 (71%)
AAS (n = 71)	4 (5.6%)	7 (9.9%)	13 (18.3%)	47 (66.2%)
Total (n = 467)	95 (20.3%)	9 (2%)	35 (7.5%)	328 (70.2%)

**Figure 1:**
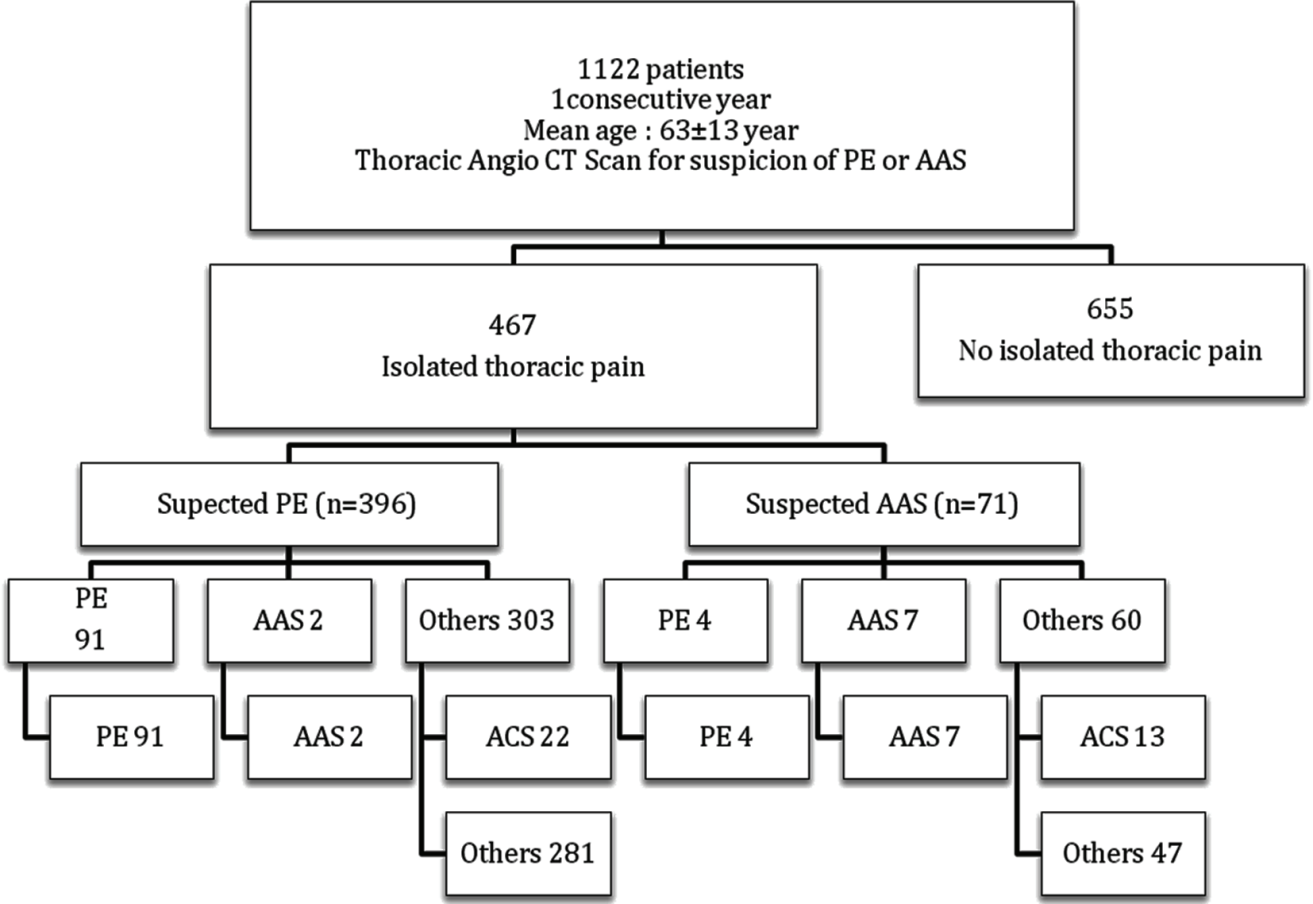
Final diagnosis among 467 patients with atypical chest pain evaluated with CTA.

The final diagnosis of the 396 patients, initially suspected of having PE, included 91 (23%) patients with PE, two (0.5%) patients with AAS, 22 (5.5%) patients with ACS and 281 (71%) patients with other diagnoses. On the other hand, among the 71 patients initially suspected of having AAS, the final diagnosis involved seven (9.9%) patients with AAS, four (5.6%) patients with PE, 13 (18.3%) patients with ACS and 47 (66.2%) patients with other diagnoses (Fig. 1, Table 1).

Thirty-five patients had a final diagnosis of ACS (22 in the pre-test PE group and 13 in the pre-test AAS group), accounting for a 7.5% of all patients presenting with atypical chest pain in the study (Table 1).

The prevalence of ACS and AAS was low in the pre-test PE group, seen in only 5.5% and 0.5% of the patients of that group respectively (P = 0.0001) (Table 2). On the other hand, the prevalence of ACS was higher (18.3%) than the prevalence of AAS (9.9%) in the pre-test AAS group (P = 0.14). In this group of patients, the prevalence of PE (5.6%) was lower than AAS (9.9%) (P = 0.34) (Table 3).

**Table 2:** Overlap Between PE, ACS and AAS Among Patients With Initial Clinical Suspicion of PE

	Prevalence of PE	Prevalence of ACS	Prevalence of AAS	P
Pre-PE (N = 396)	23%	5.5%		0.0001
	23%		0.5%	0.0001

**Table 3:** Overlap Between PE, ACS and AAS Among Patients With Initial Clinical Suspicion of AAS

	Prevalence of AAS	Prevalence of ACS	Prevalence of PE	P
Pre-AAS (N = 71)	9.9%	18.3%		0.14
	9.9%		5.6%	0.34

## Discussion

CTA has become a standard procedure in the evaluation of PE and AAS [22]. Cardiac CTA has proved to be an effective tool to rule out coronary artery disease (CAD), with a sensitivity of 93-99% and negative predictive value of 95-99% [23]. About 20% of patients with acute chest pain present to the ED with atypical acute chest pain and often require multiple examinations to exclude PE and/or AAS, in addition to excluding obstructive CAD [24].

It has been suggested that despite its high negative predictive value for CAD, cardiac CT has a limited capacity to diagnose non-cardiac causes of chest pain arising from sources outside the anatomic window it interrogates. A more comprehensive evaluation that rapidly excludes both cardiac and non-cardiac sources of chest pain should theoretically be preferable. Technical advances in cardiac CT and intravenous contrast injection protocols have made a TRO protocol feasible, which can effectively image the coronary, aortic, and pulmonary arterial beds [4, 6, 15-18]. The TRO protocol allows for simultaneous evaluation and exclusion of CAD, PE, and AAS in a single rapid test.

By potentially eliminating the need for further diagnostic testing, a TRO approach seems to improve the efficiency and downstream clinical outcomes of acute chest pain evaluation [25]. However, the implementation of TRO protocol is not without some drawbacks. Compared with standard cardiac CTA, TRO approach is associated with high radiation exposure longer breath-hold and increased contrast volume. The additional radiation exposure is attributable to the greater scan length required to image the entire pulmonary arterial circulation and thoracic aorta. In a comparative study between TRO protocol and standard CTA, it was reported that TRO protocol was associated with 46% higher radiation exposure than standard CTA [26]. Two studies showed that TRO protocol did not show any improvement of diagnostic yield, regarding ACS, PE or AAS, clinical outcomes (90 days outcome), cost of care or downstream health care resources use as compared with standard CTA [22, 25].

Another very interesting issue that should be considered when considering the implementation of TRO protocol is the incidence of ACS, PE and AAS and the overlap between the three entities in patients presenting to the ED with atypical chest pain. To our knowledge, this is the first study to show the prevalence and overlap of these three entities in a large cohort of patients. In this study, the final diagnostic yield for ACS, PE or AAS was 7.5%, 20.3% and 2% respectively. In a recent study evaluating the diagnostic yield of TRO protocol among 272 patients with acute chest pain, the prevalence of ACS, PE and AAS was 13.2%, 1.1% and 0% respectively [25]. According to the same study, the low diagnostic yield of TRO protocol for PE and AAS could be attributed to the inappropriate patient selection, including indiscriminate TRO use in patients with low pre-test probability which seemed to be driven by the clinical implications of death, disability, and litigation resulting from missed diagnosis in these cases [25].

The results of our study clearly indicate that within the pre-test PE patients, the overlap between PE (23%) on one hand and either ACS (5.5%) or AAS (0.5%) on the other hand is limited. Similarly in a small cohort of patients with high pre-test probability of PE, one study demonstrated a 21% PE yield using TRO protocol [15]. This study also demonstrated a poor overlap between PE on one hand and AAS (4%) or ACS (3%) on the other hand in their patients.

On the other hand, our study demonstrated a greater overlap between AAS and either ACS or PE among the pre-test AAS patients with the overlap being higher between AAS and ACS. This is in agreement with Yoo et al [2] who stated that AAS is much less frequent compared with ACS and there is substantial overlap in clinical symptoms and signs between ACS and AAS, ED physicians tend to mistake AAS for ACS, and the reverse also occurs. Furthermore, type A aortic dissection could be associated with coronary artery flow impairment that could mimic ACS clinical presentation.

Therefore we believe that our results are strong enough to suggest that patients presenting to ED with atypical chest pain with pre-test probability of PE should be considered for a dedicated CTA for PE only. On the other hand, those patients with a pre-test probability of AAS should be examined with either TRO protocol or a dedicated coronary CTA with extended volume of interest in the z direction to cover the entire thoracic aorta (double-rule-out protocol). A possible advantage of such latter approach is to better analyze the ascending aorta and the potential extension of the aortic dissection to the coronary arteries with ECG gated protocols.

The previously proposed algorithm might help to minimize the number of TRO protocol being performed today in favor of dedicated coronary or PE CTA. This will minimize the dose of radiation exposure and volume of contrast given to those patients as well as reduce the number of studies requiring interpretation by radiologists with special CTA experience.

Meanwhile, it also seems logical at the time being to highlight the importance of applying strict clinical criteria for patient selection rather than performing systematic TRO protocol when investigating patients with acute chest pain.

Further larger prospective randomized trials comparing these two techniques for patients presenting to ED with clinical suspicion of AAS might help to sort out this issue.

### Conclusion

The overlap between PE, AAS and ACS among patients with atypical chest pain suspected clinically of having PE is limited while the overlap between the three conditions among patients with clinical suspicion of AAS is greater and significant. Therefore, we believe that patients presenting to ED with pre-test probability of PE should be triaged for dedicated PE CTA while those with pre-test probability of AAS should be still triaged for a TRO protocol or modified cardiac CTA. The application of strict clinical criteria for the application of TRO protocol in patients with atypical chest pain is recommended.

## Abbreviations

AAS: acute aortic syndrome
ACS: acute coronary syndrome
PE: pulmonary embolism
TRO: triple rule-out
ED: emergency department
MDCT: multi-detector computed tomography
ECG: electrocardiogram
CTA: computed tomoangiography
